# Quantification and time course of subjective psychotropic and somatic effects of tetrahydrocannabinol – a prospective, single-blind, placebo-controlled exploratory trial in healthy volunteers

**DOI:** 10.1186/s12888-024-06338-2

**Published:** 2024-12-18

**Authors:** Maren Kleine-Brueggeney, Markus Huber, Lorenz Theiler, Fritz Priemer, Robert Greif

**Affiliations:** 1 Deutsches Herzzentrum der Charité (DHZC), Department of Cardiac Anesthesiology and Intensive Care Medicine, Charité - Universitätsmedizin Berlin, corporate member of Freie Universität Berlin and Humboldt-Universität zu Berlin, Berlin, Germany; 2 Statistician, Department of Anaesthesiology and Pain Medicine, Bern University Hospital, Inselspital, University of Bern, Bern, Switzerland; 3 Department of Anaesthesiology Cantonal Hospital Aarau, Aarau, Switzerland; 4 DRES. PRIEMER, Office of Medical Experts, Wonneberg, Germany; 5 University of Bern, Bern, Switzerland; 6 Department of Surgical Science, University of Torino, Torino, Italy

**Keywords:** THC, Tetrahydrocannabinol, Psychotropic effects, Somatic effects

## Abstract

**Background:**

Cannabis is increasingly used and debates about the legalisation of the recreational use of cannabis are ongoing. In this prospective, placebo-controlled study in healthy volunteers not regularly consuming cannabis, subjective psychotropic and somatic effects after a single dose of intravenous THC were assessed and quantified over 48 h.

**Methods:**

Twenty-five healthy volunteers received a single IV bolus of THC and 6 received normal saline. Psychotropic and somatic effects of THC were assessed by two questionnaires that were completed at up to 14 timepoints from shortly before drug administration to 48 h later.

**Results:**

Demographic data did not differ between groups. Differences between THC and placebo for all assessed effects, except for euphoria, irritation and headache, were clearly discernible. Subdimensions related to positive mood were less and those related to negative mood were more pronounced in the THC group. Peak plasma concentrations were observed at 1 to 5 min after THC administration while peak effects occurred between 45 and 60 min. Differences between THC and placebo were pronounced and seen for up to 90 to 120 min for most effects, except for “sleepiness” and “deactivation”, where the effect of THC was discernible for up to 5 h. At 24 and 48 h, there were no statistically significant difference between THC and placebo group.

**Conclusions:**

THC triggers a large range of psychotropic and somatic effects with peak effects at 45 to 60 min after IV administration of THC, much later than plasma peak levels. Most effects are short-lasting with a duration of up to 2 h, but some effects like sleepiness and deactivation can be longer-lasting and persist for 5 h or longer in cannabis-naïve or cannabis-abstinent individuals. Since effects of THC demonstrate a time course that differs from the time course of plasma concentrations it might be important to base the judgment of a possible impairment related to THC consumption on clinical or behavioral tests in addition to THC plasma levels.

**Trial registration:**

www.isrctn.com; registration number ISRCTN53019164.

**Supplementary Information:**

The online version contains supplementary material available at 10.1186/s12888-024-06338-2.

## Background

Cannabis consumption is increasing and debates about the legalisation of the recreational use of cannabis are ongoing in numerous countries. Medical applications of cannabis and its active compounds delta-9-tetrahydrocannabinol (d-9-THC or THC) and cannabidiol have been studied [1–5], particularly for control of pain [1, 6, 7], spasticity [8–10], neurological [11–13] as well as gastrointestinal disorders [14] and nausea and vomiting [15, 16]. Similarly, subjective effects of THC have increasingly become a focus of academic interest [17, 18].

THC is the major intoxicating component of cannabis species. It is metabolised to 11-hydroxy-delta-9-tetrahydrocannabinol (11-OH-THC) and to 11-nor-9-carboxy-delta-9-tetrahydrocannabinol (11-COOH-THC) by hepatic cytochrome P450 (CYP) mediated hydroxylation [19]. The main cytochrome involved in this process is CYP2C9, with CYP2C19 and CYP3A4 playing a subordinated role [20]. 11-OH-THC is recognised as a strong psychoactive metabolite of THC, while 11-COOH-THC is reported to be non-psychotropic [21].

The effects of THC consumption appear to depend on various factors such as the mode of consumption, the dose, and the frequency of consumption. Plasma concentrations after oral consumption are lower compared to inhalational administration and the effects depend on the individual degree of absorption and first pass effect. The effects of inhalation have been reported to vary depending on the inhalation technique [17]. A study on neurocognitive performance demonstrated significant differences in impairment of neurocognitive performance between heavy and occasional cannabis users, with occasional users demonstrating a higher degree of impairment [22].

In this prospective controlled study in healthy volunteers not regularly consuming cannabis, subjective psychotropic and somatic effects after a single injection of intravenous THC were assessed and quantified over a time course of 48 h.

## Methods

This is a prospective, single-blind, placebo-controlled, exploratory trial assessing the subjective psychotropic and somatic effects of intravenous THC and its metabolites in healthy volunteers not regularly consuming cannabis. The study was performed at the Department of Anaesthesiology and Pain Medicine of the Bern University Hospital in Switzerland with ethics committee approval (Cantonal Ethics Committee Bern, approval number KEK 241–09) and approval of the relevant authorities (Federal Office of Public Health of the Swiss Confederation, Swissmedic). It was registered at www.isrctn.com (registration number ISRCTN53019164, registered 14.04.2010). All volunteers gave written informed consent prior to enrollment. This study was part of a larger trial on pharmacokinetics of intravenous THC with enrollment from 07/10 to 02/11 [23].

The study aimed to characterize and quantify the subjective psychotropic and somatic effects of THC and its metabolites and their time-course after a single IV bolus of THC. Secondary aims were to describe differences in the psychotropic and somatic state between volunteers receiving THC or placebo, and to define a time at which effects become negligible, defined as no difference between the THC and the placebo group.

### Volunteers

Inclusion criteria were age over 18 years and volunteers who had either never smoked tobacco or cannabis, or had not smoked for at least three months and had not consumed cannabis for at least one month. Exclusion criteria were pregnancy (test performed in females), body mass index (BMI) < 16 or > 35 kg m^− 2^, suspected ischemic heart disease, cardiac arrhythmias, hepatic cytochrome P450 activity altering medication, treated or suspected psychiatric disease at any point during lifetime, and any illegal substance use.

### Allocation, blinding and study setting

Since cytochrome P 2C9 (CYP2C9) is key in degradation of THC, volunteers (*n* = 306), recruited from the Medical School of the University of Bern, were screened for single nucleotide polymorphisms (SNPs) of CYP2C9 (*2: Arg144Cys and *3: Ile359Leu) and classified by genotype. These SNPs were chosen as they were the first polymorphisms of CYP2C9 to be discovered and belong to the most common SNPs of CYP2C9 in a Caucasian population [24]. All volunteers expressing SNPs (heterozygous or homozygous, *n* = 20) and selected wild type carriers (*n* = 5) were included in the study (total *n* = 25), receiving THC. This was done to characterise pharmacokinetics of IV THC in view of possible genetic influences of the SNPs on THC degradation as published earlier [23]. Additionally, selected wild-type carriers were included to receive placebo (*n* = 6). These volunteers were selected based on availability. Volunteers, but not study personnel, were blinded about their allocation to receive THC or placebo, by using visually identical vials containing the study drug and labelled by a code that was only identifiable to the study personnel (THC or placebo).

In brief, volunteers were monitored in one of the department’s postoperative care units (PACU) that was dedicated solely to the study. Monitoring included continuous non-invasive ECG and SpO_2_, and invasive blood pressure measurements. All volunteers had a patent intravenous line throughout their stay in PACU, and an arterial line for blood sampling and blood pressure monitoring. The study drug was administered between 9 and 10 AM and consisted of a single IV bolus of 0.1 mg/kg (0.32 µM/kg) THC or an equivalent volume of NaCl 0.9%. THC was provided by THCPharma (Frankfurt am Main, Germany) and the IV THC injection solution was prepared by the hospital pharmacy of the University Hospital Bern, Inselspital, Bern, Switzerland according to the method described by Naef [25]. We decided to use IV THC and not an oral or inhaled preparation to avoid any bias of bioavailability or differences in dosing resulting from smoking technique.

### Measurements

Demographic data of all volunteers such as age, sex, American Society of Anesthesiologists’ (ASA) physical status classification, weight and height were recorded. Plasma levels of THC, 11-OH-THC and 11-COOH-THC were measured by liquid chromatography with tandem mass spectrometry before and in intervals at 1, 2, 5, 10, 15, 20, 30, 45, 90, 180, 300 min and at 24 and 48 h after the IV bolus to establish a dedicated pharmacokinetic model of IV THC [23]. Levels of endocannabinoids were measured to examine the influence of THC administration on endogenous cannabinoid levels and signalling [26]. In essence, pharmacokinetic modelling revealed no impact of the CYP2C9 status on THC or 11-OH-THC plasma levels after IV administration, but a possible reduced exposure to 11-COOH-THC for subjects homozygous for CYP2C9*3 [23].

To assess and quantify psychotropic and somatic effects of THC and its metabolites over time two questionnaires were used:

#### ***Questionnaire 1***

was adapted from publications by Naef [1, 25] and focused on psychotropic and somatic effects of THC employing a visual analogue scale from 0 to 10 on 15 items (0 = minimum, 10 = maximum). The items were: (1) Sleepiness; (2) euphoria; (3) irritation; (4) anxiety; (5) tenseness and aggressiveness; (6) confusion and disorientation; (7) change of inner perception; (8) change of outer perception; (9) hallucinations; (10) strange thoughts/ ideas/ mood; 11) headache; 12) difficulties in breathing; 13) cardiac problems such as fast pulse; 14) dry mouth; and 15) nausea.

#### ***Questionnaire 2***

was the German multidimensional Self-Report Mood Rating Inventory (BSKE 30) by Janke et al. [27], used for the assessment of mood state. It contains 28 items on the mood state plus 2 items on physical well-being, each described by a noun and two related adjectives. Each item is rated on a 7-point Likert scale (0 = not at all to 6 = very much). Items are summarized into 17 subdimensions (balance; lifted mood; activation; confidence; capability; vitality; hedonic reactivity; extraversion; excitation; bad mood; irritation; anxiety; depression; deactivation; anhedonic reactivity, introversion; and physical well-being).

Questionnaire 1 was completed before administration of THC and at 10, 30, 45, 60, 75, 90, 120, 150, 180, 240, 300 min after administration and after 24 and after 48 h (14 timepoints). Questionnaire 2 was completed before administration of THC and at 30, 90, 150 and 300 min after administration and after 24 and 48 h (7 timepoints).

### Statistical considerations

#### ***Sample size***

For this exploratory study, sample size was not determined by statistical means, but by practical aspects: As described above, all screened volunteers expressing SNPs (*n* = 20) and selected wild type carriers (*n* = 5) were included with the aim to characterise pharmacokinetics of IV THC. For *2/ *3, respectively, this included 6 heterozygous/wild type, 3 homozygous/ wild type, 5 wild type/ heterozygous, 3 wild type/ homozygous, and 3 heterozygous/ heterozygous individuals. Six volunteers received placebo. This was also what we deemed feasible in our setting.

#### Summary statistics

Categorical variables were summarized by counts and percentages and group comparisons are based on the chi-square test or the exact Fisher test when expected counts in some cells were lower than 5. Quantitative variables were summarized with mean and standard deviation for normally distributed variables and with median and interquartile range otherwise. Group comparisons of quantitative variables were based on Student’s t-test in case of normally distributed variables and the unpaired two-samples Wilcoxon test otherwise.

#### Statistical analysis of questionnaires

For questionnaire 1, individual categories as well as pooled psychotropic and somatic variables were analyzed. As detailed in the related validated questionnaire instructions, questionnaire 2 was analyzed within the predefined 17 subdimensions. For both questionnaires, summary statistics at each timepoint are presented with median and interquartile range. In terms of group comparisons between the volunteers who received THC versus the placebo group, a generalized linear mixed model (GLMM) with a beta distribution for the outcome was applied. A random offset for each volunteer accounted for the repeated measure design of the study. To account for the bounded nature of the outcome, for example the value range between 0 and 10 in case of a response item of questionnaire 1, the response items were transformed to the (0,1) interval by means of a linear transformation suggested by Smithson and Verkuilen [28], thus allowing the outcomes to be analyzed by means of a beta distribution. Analysis was performed as a complete-case analysis. Additionally, a sensitivity analysis was performed where missing values were imputed using the timepoint-wise median value. The p-values derived from the group comparisons at each timepoint were adjusted for multiple comparison by means of the Bonferroni correction.

#### Statistical analysis of CYP2C9*3 dependency

The 22 volunteers receiving THC and not homozygous for CYP2C9*3 were compared to the 3 volunteers receiving THC and being homozygous for CYP2C9*3. A generalized additive model (GAM) through the data points was used to assess the peak. Due to the small sample of only 3 volunteers homozygous for *3, no further analysis for statistical significance was performed as this was deemed inadequate.

#### ***Statistical software***

All statistical analyses were performed using R (R Core Team (2020). R: A language and environment for statistical computing. R Foundation for Statistical Computing, Vienna, Austria. URL https://www.R-project.org/). A *p*-value of < 0.05 was deemed statistically significant.

## Results

A total of 25 volunteers received THC and 6 volunteers received placebo and completed the study. Demographic data of the volunteers in both cohorts were comparable (Table 1).

**Table 1:** Demographic data of the volunteers. Data are number (%), median [IQR], or mean (SD). WT: wild type; het: heterozygous; hom: homozygous; BMI: body mass index; ASA: American Society of Anesthesiologists; ASA class I refers to a normal healthy person with no systemic disease; THC: delta-9-tetrahydrocannabinol

	Placebo	THC	*P*
	*n* = 6	*n* = 25	
**Sex** (female)	2 (33.3%)	14 (56.0%)	0.394
**Age** (years)	22.5 [22.0;23.8]	23.0 [21.0;25.0]	0.980
**Weight** (kg)	70.0 [65.0;79.5]	65.0 [57.0;73.0]	0.305
**Height** (cm)	176 (12.3)	173 (8.90)	0.609
**BMI** (kg.m^− 2^)	23.6 (3.78)	22.5 (3.25)	0.531
**ASA** class I	6 (100%)	25 (100%)	-
**THC dosage** (mg)	-	6.50 [5.70;7.30]	-
Genotype *2:			
WT/ het/ hom	6/ 0/ 0 (100/ 0/ 0%)	13/ 9/ 3 (52/ 36/ 12%)	0.168
Genotype 3*:			
WT/ het/ hom	6/ 0/ 0 (100/ 0/ 0%)	14/ 8/ 3 (56/ 32/ 12%)	0.209

### Psychotropic and somatic effects of THC and its metabolites

Questionnaires were filled in by the volunteers for the specified time points. There was a total of 187 missing data points for questionnaire 1 (2.9% of data points), and a total of 205 missing data points for questionnaire 2 (3.1% of data points).

**Fig. 1:**
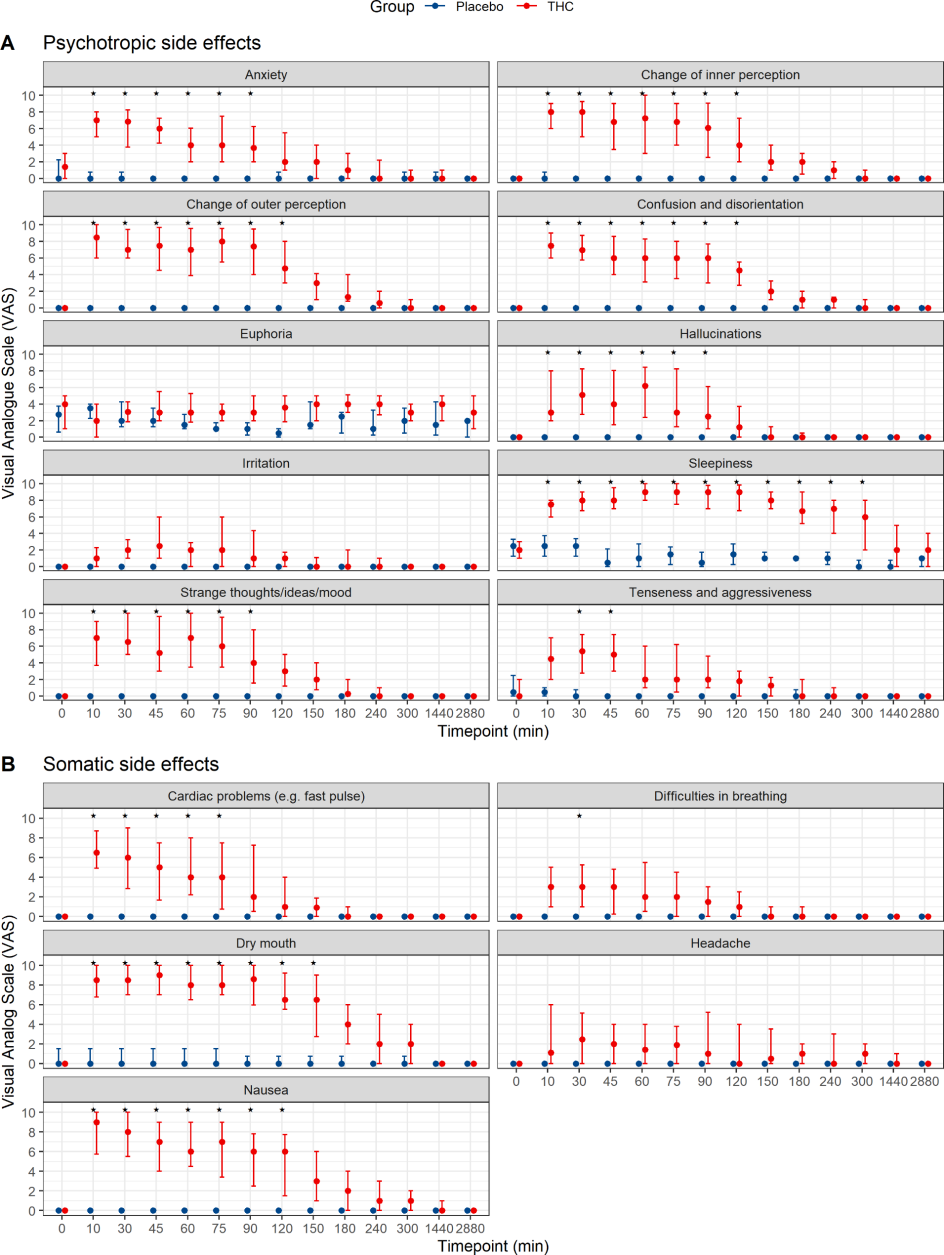
Psychotropic **(A)** and somatic **(B)** effects as assessed by questionnaire 1. Data are median and IQR. The asterisks (*) mark all statistically significant differences between placebo and THC groups

The results for the psychotropic and somatic effects over time as assessed by questionnaire 1 are given in Fig. 1. Differences between THC and placebo for all assessed effects, except for euphoria, irritation and headache, are clearly visible. Detailed results for all items are summarized in supplementary table 1A and 1B. Differences between THC and placebo were pronounced and seen for up to 120 min for most effects, except for “sleepiness”, where an effect of THC was discernible for up to 6 h.

**Fig. 2:**
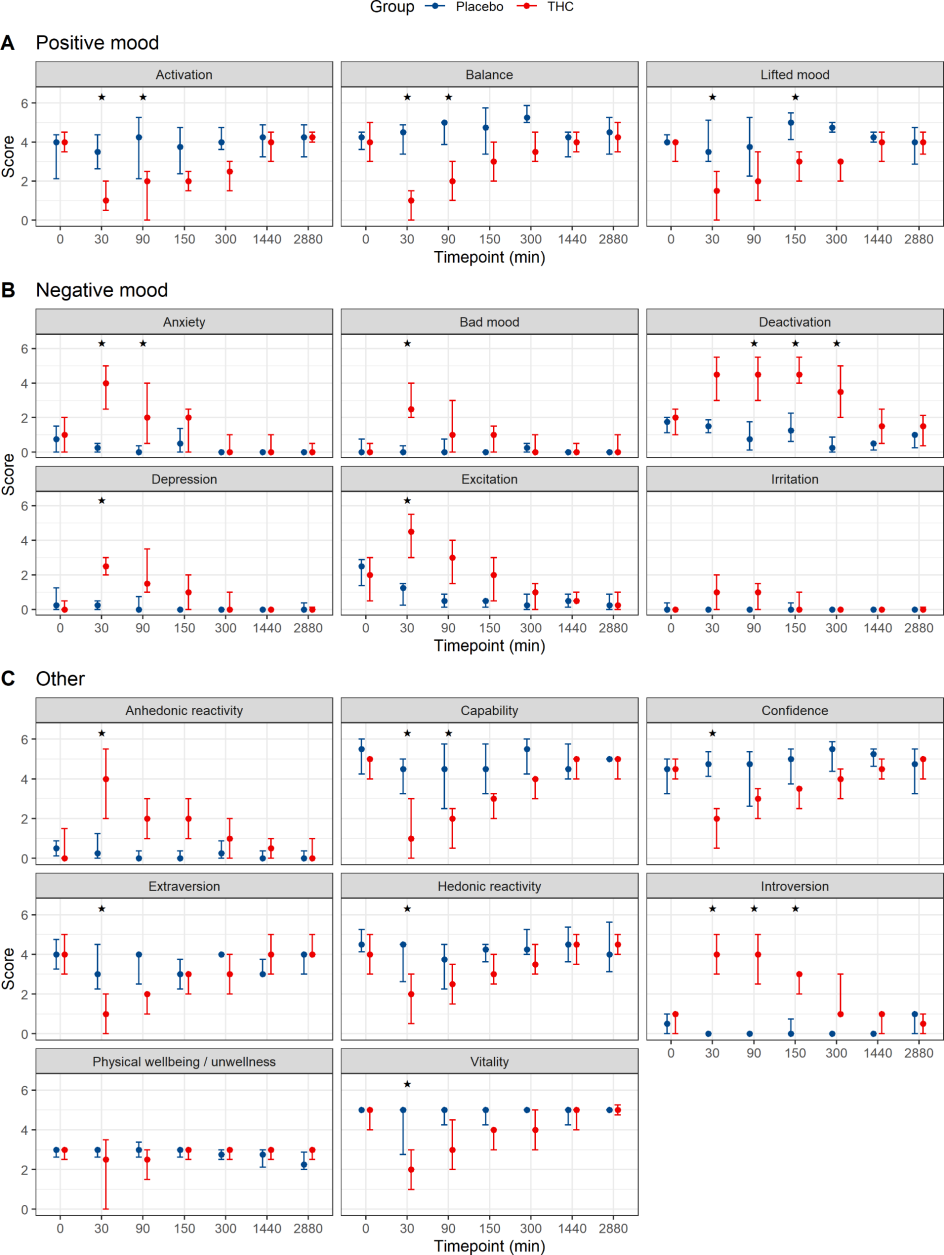
Effects of IV THC as assessed by questionnaire 2, summarized by the different subdimensions of the global dimensions “positive mood” **(A)**, “negative mood” **(B)** and “other” **(C)**. Data are median and IQR. The asterisks (*) mark all statistically significant differences between placebo and THC groups

**Fig. 3:**
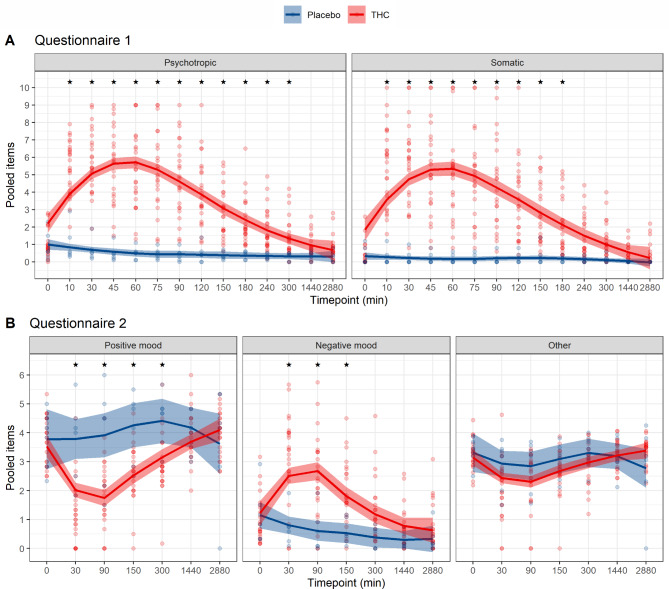
Global dimensions of psychotropic and somatic effects to assess peak effect and duration of effects in questionnaire 1 (**A**) and questionnaire 2 (**B**). Peak effects occur between 45 and 90 min after THC injection. After 6 h, no statistically significant difference between the THC and placebo group is discernible. Asterisks (*)are used to mark all statistically significant differences

The effects as assessed by questionnaire 2 are graphically summarized in Fig. 2. Similar to the results of questionnaire 1, albeit somewhat less pronounced, significant differences were found for most items between the THC and the placebo group and differences were most pronounced in the first 90 min after THC injection. In general, subdimensions related to positive mood were less reported in the THC group and subdimensions related to negative mood were more intense in the THC group. This can also be seen in Fig. 3. Detailed results of questionnaire 2 are summarized in supplementary Table 2. No adverse events or harms were observed.

### Peak effect and duration of effects

**Fig. 4:**
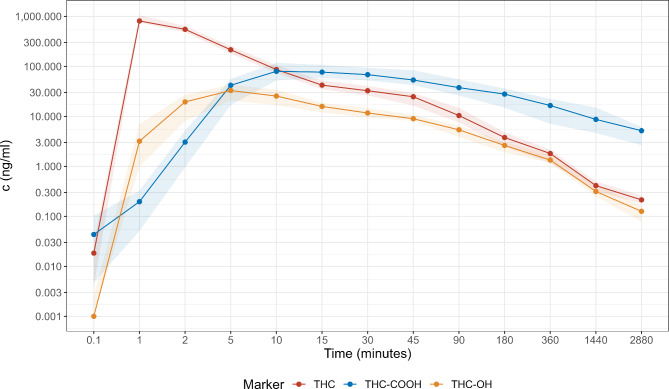
Plasma concentrations of THC, 11-OH-THC and 11-COOH-THC over time in volunteers after a single IV injection of THC. Data are median and interquartile range

To assess the time of the peak effects and the duration of the effects, items were pooled: For questionnaire 1, items were pooled into the categories psychotropic effects and somatic effects. For questionnaire 2, items were pooled into the global dimensions positive mood, negative mood, and other, as described by the developers of the questionnaire (Fig. 3). It can be appreciated that peak effects occur between 45 and 60 min. Some effects persisted for up to 5 h. After 24 h, no statistically significant difference between THC and placebo group is discernible. For visual comparison of the timing of peak plasma concentrations and peak effects, plasma concentrations of THC, 11-OH-THC and 11-COOH-THC over time for volunteers receiving THC are given in Fig. 4. Peak plasma levels of THC occurred at 1 min and peak plasma levels of 11-OH-THC occurred at 5 min after administration, much earlier than peak effects.

There were no missing data for the plasma concentrations. For the data from the two questionnaires the missing data rate was 2.6%. A sensitivity analysis with imputation of missing values demonstrated robustness of the results. The sensitivity analysis is shown in Supplementary Fig. 1 to 4.

### Comparison of volunteers not homozygous for CYP2C9*3 with volunteers homozygous for CYP2C9*3

**Fig. 5:**
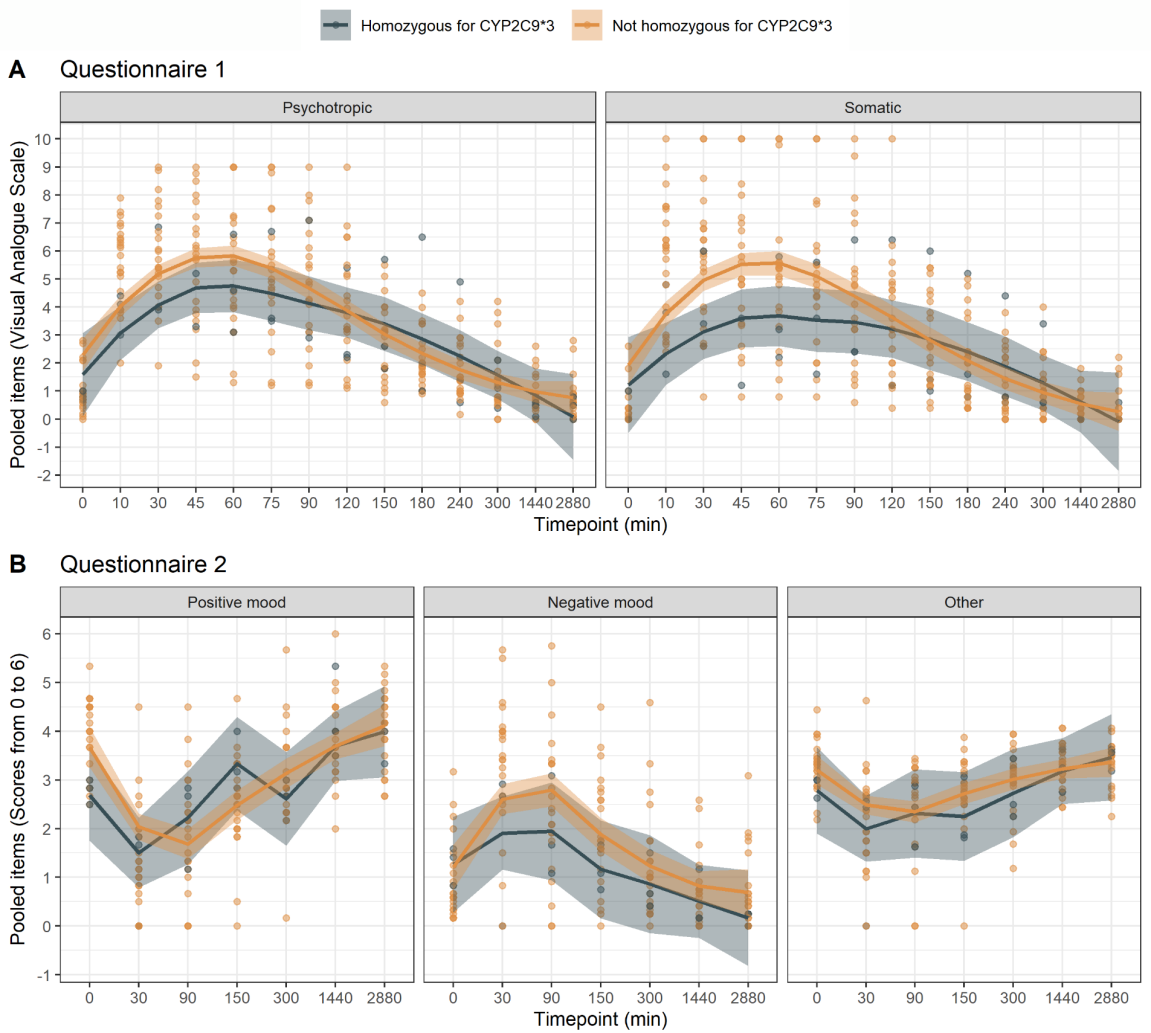
Graphical analysis of THC effects in volunteers homozygous (*n* = 3) or not homozygous (*n* = 22) for CYP2C9*3, receiving IV THC. No statistical significance testing was performed due to the small sample size

The graphical analysis of THC effects in volunteers not homozygous for CYP2C9*3 compared to volunteers homozygous for CYP2C9*3 (Fig. 5) suggests that volunteers not homozygous for *3 might demonstrate somewhat more pronounced effects, but due to the small sample size testing for statistical significance was not performed.

## Discussion

This prospective, placebo-controlled study evaluated the psychotropic and somatic effects of IV THC in healthy volunteers over 48 h. Significant and strong effects of THC were found, most of which lasted for roughly 2 h, with some effects discernible for up to 5 h. The effects started almost immediately with peak effects between 45 and 60 min after IV injection. Effects comprised a large range of psychotropic and somatic effects. The most pronounced psychotropic effects were anxiety, change of inner and outer perception, confusion and disorientation, hallucinations, strange thoughts/ ideas/ mood, and sleepiness.

These results are in line with previous studies investigating inhaled THC which showed that psychotropic effects occurred almost immediately with a peak effect after 15 to 30 min and declining over 3 h [29]. Previous results also postulated that plasma concentrations correlate poorly with the extent of early psychoactive effects since peak effects occur when plasma concentrations are already declining [21, 30]. Although it was not the principal aim of this study to correlate psychotropic effects to THC plasma levels, our data on IV THC are in line with these earlier findings from inhaled THC: Peak plasma levels of THC in our sample occurred at 1 min after injection and peak plasma levels of 11-OH-THC occurred at 5 min after injection (Fig. 4), hence much earlier than peak psychotropic and somatic effects [23]. This is likely related to the distribution of THC from blood to its effect site, the central nervous system, during the rapid distribution phase of THC kinetics [29]. In the following elimination phase of THC, where concentrations of THC and effects have been described to decline in parallel, plasma levels of THC might be more predictive of psychoactive effects [29, 31].

With regards to the duration of effects, the presented data demonstrate that many effects are rather short-lasting with most differences detected for up to 2 h, and only few effects differing after 5 h. Some effects, such as sleepiness and deactivation, persist longer than others. In general, the effects of THC consumption on neurocognitive impairment are likely influenced by the frequency of consumption [22]. With regards to the duration of effects in infrequent cannabis users, others have recently published that the effects of inhalational cannabis consumption lasted for up to six hours on average, similar to our results [32]. Previous data from regular cannabis users demonstrated THC effects in the sense of an impaired fitness to drive for up to 4.5 h after ad libitum smoked THC [33].

The present study did not assess driving ability or other aspects linked to liability which might be of interest when debating the legalisation of THC use. However, since the time course of somatic and psychotropic effects of THC differs from the time course of plasma concentrations of THC and its metabolites, measurements of cognitive or behavioural drug effects might be valuable additions the measurement of drug concentrations to determine a possible impairment resulting from THC consumption. In line with this, others have recently assessed methods to detect cannabis impairment such as the DRUID^®^ test and field sobriety test [34].

Regarding the effects of the THC metabolites, it has been reported that 11-OH-THC is the major intoxicating metabolite of THC, while 11-COOH-THC is reported to be non-psychotropic [21]. It has, however, been suggested that 11-COOH-THC might have other effects, such as anti-inflammatory and analgesic effects [29, 35, 36] as well as effects on sensations like itching, burning and deep pain [21]. Our sample included three individuals homozygous for CYP2C9*3, who could have a reduced exposure to 11-COOH-THC [23]. Since 11-COOH-THC is described to be non-psychotropic [21] the primary analysis of this study was done including all volunteers receiving THC compared to all volunteers receiving placebo. To assess a possible effect of CYP2C9*3 on THC effects, we compared the 22 volunteers receiving THC and not homozygous for CYP2C9*3 to the 3 volunteers receiving THC and being homozygous for CYP2C9*3. Graphical analysis demonstrated possibly increased effects of IV THC in individuals homozygous for *3, which could suggest an anti-psychotropic effect of 11-COOH-THC, if any. Given the small sample size this remains speculative and further investigations on the effects of 11-COOH-THC are needed.

### Limitations

The present study involved a small and uneven sample size of only 25 volunteers receiving THC and 6 volunteers receiving placebo and was, with its exploratory design, not powered to detect differences for each assessed item over the entire study period. This was due to the design of the pharmacokinetic study that was conducted in parallel to the present study [23]. While the effects of THC could be clearly characterized, the small sample size prohibits further and more detailed analyses regarding the relationship between plasma levels and effect, effects of the different metabolites or subgroup analyses for the various genotypes. Secondly, some effects persisted at 5 h after THC administration. Since effects were not assessed between 5 and 24 h after injection the exact duration of effects between 5 and 24 h remains unclear. Lastly, volunteers were all cannabis-naïve or –abstinent and THC was given intravenously. Effects could well differ in regular consumers or with different routes of administration.

## Conclusion

THC triggers a large range of psychotropic and somatic effects with peak effects at 45 to 60 min after IV administration of THC. Effects might vary between cannabis-naïve or regular users and the time course of effects differs from the time course of plasma levels, which peak much earlier than the psychoactive and somatic effects. Effects like anxiety, change of inner and outer perception, confusion and disorientation, hallucinations, strange thoughts/ ideas/ mood, and sleepiness were most pronounced. Many effects are short-lasting with a duration of up to 2 h, but some effects like sleepiness and deactivation can be longer lasting and persist for 5 h or longer in cannabis-naïve or cannabis-abstinent individuals. Hence, assessment of a possible impairment resulting from THC consumption should likely include not only THC plasma levels, but also results of clinical tests that assess the actual psychotropic or somatic effects of THC in an individual.

## Electronic supplementary material

Below is the link to the electronic supplementary material.

Supplementary Material 1

Supplementary Material 2: Fig. 1: Psychotropic (A) and somatic (B) effects as assessed by questionnaire 1. Data are median and IQR. Missing values are imputed using the timepoint-wise median value. The asterisks (*) mark all statistically significant differences between placebo and THC groups.

Supplementary Material 3: Fig. 2: Effects of IV THC as assessed by questionnaire 2, summarized by the different subdimensions of the global dimensions “positive mood” (A), “negative mood” (B) and “other” (C). Data are median and IQR. Missing values are imputed using the timepoint-wise median value. The asterisks (*) mark all statistically significant differences between placebo and THC groups.

Supplementary Material 4: Fig. 3: Global dimensions of psychotropic and somatic effects to assess peak effect and duration of effects. Missing values are imputed using the timepoint-wise median value. Asterisks (*) are used to mark all statistically significant differences.

Supplementary Material 5: Fig. 4: Graphical analysis of THC effects in volunteers homozygous (*n* = 3) or not homozygous (*n* = 22) for CYP2C9*3, receiving IV THC. Missing values are imputed using the timepoint-wise median value. No statistical significance testing was performed due to the small sample size.

## Data Availability

The datasets used and/or analysed during the current study are available from the corresponding author on reasonable request and with ethics committee approval and approval of the relevant authorities.

## Abbreviations

ASA: American Society of Anesthesiologists
CYP: hepatic cytochrome P450
d: 9-THC-delta-9-tetrahydrocannabinol
GAM: Generalized additive model
IV: Intravenous
PACU: Postoperative care unit
SNP: Single nucleotide polymorphism
THC: Tetrahydrocannabinol
11: COOH-THC-11-nor-9-carboxy-delta-9-tetrahydrocannabinol
11: OH-THC-11-hydroxy-delta-9-tetrahydrocannabinol
