# Supplementary material for: Quantification and time course of subjective psychotropic and somatic effects of tetrahydrocannabinol – a prospective, single-blind, placebo-controlled exploratory trial in healthy volunteers
Source: BMC Psychiatry. 2024 Dec 18;24:902. doi: 10.1186/s12888-024-06338-2 (PMC11654089; doi:10.1186/s12888-024-06338-2)
Supplement: Supplementary file 1 — Supplementary Material 1 [file 12888_2024_6338_MOESM1_ESM.docx]

Supplementary Table 1A: Psychotropic effects of THC as assessed by questionnaire 1 over time. Data represent measurements on a visual analog scale from 0 to 10 and are expressed as median (IQR).

|  | 0 min | 10 min | 30 min | 45 min | 60 min | 75 min | 90 min | 120 min | 150 min | 180 min | 240 min | 300 min | 1440 min (24h) | 2880 min (48h) |
| --- | --- | --- | --- | --- | --- | --- | --- | --- | --- | --- | --- | --- | --- | --- |
| **Pooled psychotropic effects** | | | | | | | | | | | | | | |
| Placebo | 0.6 [0.4 - 1.3] | 0.5 [0.4 - 1.2] | 0.5 [0.3 - 0.7] | 0.4 [0.2 - 0.5] | 0.5 [0.2 - 0.6] | 0.4 [0.2 - 0.6] | 0.4 [0.2 - 0.5] | 0.4 [0.1 - 0.5] | 0.3 [0.2 - 0.5] | 0.4 [0.2 - 0.6] | 0.3 [0.2 - 0.4] | 0.4 [0.1 - 0.5] | 0.4 [0.1 - 0.6] | 0.3 [0.0 - 0.4] |
| THC | 0.8 [0.6 - 1.0] | 5.4 [4.1 - 6.5] | 5.6 [4.6 - 7.0] | 5.8 [3.8 - 7.0] | 5.3 [3.9 - 6.7] | 5.0 [3.9 - 6.6] | 4.8 [3.1 - 6.4] | 3.5 [2.7 - 5.2] | 2.8 [1.8 - 3.8] | 1.9 [1.6 - 2.7] | 1.4 [1.0 - 2.6] | 1.2 [0.7 - 1.7] | 0.7 [0.5 - 0.9] | 0.6 [0.4 - 0.9] |
| P | 1.00 | <0.001 | <0.001 | <0.001 | <0.001 | <0.001 | <0.001 | <0.001 | <0.001 | <0.001 | <0.001 | 0.045 | 1.00 | 1.00 |
| **Anxiety** | | | | | | | | | | | | | | |
| Placebo | 0.0 [0.0 - 2.3] | 0.0 [0.0 - 0.8] | 0.0 [0.0 - 0.7] | 0.0 [0.0 - 0.0] | 0.0 [0.0 - 0.0] | 0.0 [0.0 - 0.0] | 0.0 [0.0 - 0.0] | 0.0 [0.0 - 0.8] | 0.0 [0.0 - 0.0] | 0.0 [0.0 - 0.0] | 0.0 [0.0 - 0.0] | 0.0 [0.0 - 0.8] | 0.0 [0.0 - 0.7] | 0.0 [0.0 - 0.0] |
| THC | 1.4 [0.0 - 3.0] | 7.0 [5.0 - 8.0] | 6.8 [3.7 - 8.3] | 6.0 [4.2 - 7.3] | 4.0 [2.0 - 6.1] | 4.0 [2.0 - 7.5] | 3.7 [2.0 - 6.2] | 2.0 [1.0 - 5.5] | 2.0 [0.0 - 4.0] | 1.0 [0.0 - 3.0] | 0.0 [0.0 - 2.2] | 0.0 [0.0 - 1.0] | 0.0 [0.0 - 1.0] | 0.0 [0.0 - 0.0] |
| P | 1.00 | <0.001 | <0.001 | <0.001 | 0.004 | 0.001 | 0.033 | 0.781 | 1.00 | 1.00 | 1.00 | 1.00 | 1.00 | 1.00 |
| **Change of inner perception** | | | | | | | | | | | | | | |
| Placebo | 0.0 [0.0 - 0.0] | 0.0 [0.0 - 0.7] | 0.0 [0.0 - 0.0] | 0.0 [0.0 - 0.0] | 0.0 [0.0 - 0.0] | 0.0 [0.0 - 0.0] | 0.0 [0.0 - 0.0] | 0.0 [0.0 - 0.0] | 0.0 [0.0 - 0.0] | 0.0 [0.0 - 0.0] | 0.0 [0.0 - 0.0] | 0.0 [0.0 - 0.0] | 0.0 [0.0 - 0.0] | 0.0 [0.0 - 0.0] |
| THC | 0.0 [0.0 - 0.0] | 8.0 [6.0 - 9.0] | 8.0 [5.0 - 9.2] | 6.8 [3.5 - 9.0] | 7.2 [3.0 - 10.0] | 6.8 [4.0 - 9.0] | 6.1 [2.5 - 9.1] | 4.0 [2.0 - 7.3] | 2.0 [1.0 - 4.0] | 2.0 [0.5 - 3.0] | 1.0 [0.0 - 2.0] | 0.0 [0.0 - 1.0] | 0.0 [0.0 - 0.0] | 0.0 [0.0 - 0.0] |
| P | 1.00 | <0.001 | <0.001 | <0.001 | <0.001 | <0.001 | <0.001 | <0.001 | 1.00 | 1.00 | 1.00 | 1.00 | 1.00 | 1.00 |
| **Change of outer perception** | | | | | | | | | | | | | | |
| Placebo | 0.0 [0.0 - 0.0] | 0.0 [0.0 - 0.0] | 0.0 [0.0 - 0.0] | 0.0 [0.0 - 0.0] | 0.0 [0.0 - 0.0] | 0.0 [0.0 - 0.0] | 0.0 [0.0 - 0.0] | 0.0 [0.0 - 0.0] | 0.0 [0.0 - 0.0] | 0.0 [0.0 - 0.0] | 0.0 [0.0 - 0.0] | 0.0 [0.0 - 0.0] | 0.0 [0.0 - 0.0] | 0.0 [0.0 - 0.0] |
| THC | 0.0 [0.0 - 0.0] | 8.5 [6.0 - 10.0] | 7.0 [6.0 - 9.4] | 7.5 [4.5 - 9.7] | 7.0 [3.9 - 9.6] | 8.0 [5.5 - 9.6] | 7.4 [4.0 - 9.5] | 4.7 [3.0 - 8.0] | 3.0 [1.0 - 4.1] | 1.3 [0.8 - 4.0] | 0.6 [0.0 - 2.0] | 0.0 [0.0 - 1.0] | 0.0 [0.0 - 0.0] | 0.0 [0.0 - 0.0] |
| P | 1.00 | <0.001 | <0.001 | <0.001 | <0.001 | <0.001 | <0.001 | <0.001 | 0.697 | 1.00 | 1.00 | 1.00 | 1.00 | 1.00 |
| **Confusion and disorientation** | | | | | | | | | | | | | | |
| Placebo | 0.0 [0.0 - 0.0] | 0.0 [0.0 - 0.0] | 0.0 [0.0 - 0.0] | 0.0 [0.0 - 0.0] | 0.0 [0.0 - 0.0] | 0.0 [0.0 - 0.0] | 0.0 [0.0 - 0.0] | 0.0 [0.0 - 0.0] | 0.0 [0.0 - 0.0] | 0.0 [0.0 - 0.0] | 0.0 [0.0 - 0.0] | 0.0 [0.0 - 0.0] | 0.0 [0.0 - 0.0] | 0.0 [0.0 - 0.0] |
| THC | 0.0 [0.0 - 0.0] | 7.5 [6.0 - 9.0] | 7.0 [5.7 - 8.7] | 6.0 [4.0 - 8.6] | 6.0 [3.1 - 8.3] | 6.0 [3.5 - 8.0] | 6.0 [3.0 - 7.7] | 4.5 [2.7 - 5.5] | 2.0 [1.0 - 3.3] | 1.0 [0.0 - 2.0] | 1.0 [0.0 - 1.3] | 0.0 [0.0 - 1.0] | 0.0 [0.0 - 0.0] | 0.0 [0.0 - 0.0] |
| P | 1.00 | <0.001 | <0.001 | <0.001 | <0.001 | <0.001 | <0.001 | <0.001 | 0.754 | 1.00 | 1.00 | 1.00 | 1.00 | 1.00 |
| **Euphoria** | | | | | | | | | | | | | | |
| Placebo | 2.8 [0.6 - 3.8] | 3.5 [2.2 - 4.0] | 2.0 [1.3 - 4.2] | 2.0 [1.2 - 3.5] | 1.5 [1.0 - 2.8] | 1.0 [1.0 - 1.8] | 1.0 [0.2 - 1.8] | 0.5 [0.0 - 1.0] | 1.5 [1.0 - 4.2] | 2.5 [0.5 - 3.0] | 1.0 [0.3 - 3.2] | 2.0 [0.5 - 3.5] | 1.5 [0.3 - 4.2] | 2.0 [0.0 - 2.0] |
| THC | 4.0 [1.0 - 5.0] | 2.0 [0.0 - 4.0] | 3.0 [1.9 - 4.3] | 3.0 [2.0 - 5.5] | 3.0 [1.8 - 5.3] | 3.0 [2.0 - 4.0] | 3.0 [2.0 - 5.0] | 3.6 [1.8 - 5.0] | 4.0 [2.0 - 5.0] | 4.0 [3.0 - 5.1] | 4.0 [2.7 - 5.0] | 3.0 [2.0 - 4.0] | 4.0 [2.0 - 5.0] | 3.0 [1.0 - 5.0] |
| P | 1.00 | 1.00 | 1.00 | 1.00 | 1.00 | 1.00 | 1.00 | 1.00 | 1.00 | 1.00 | 1.00 | 1.00 | 1.00 | 1.00 |
| **Hallucinations** | | | | | | | | | | | | | | |
| Placebo | 0.0 [0.0 - 0.0] | 0.0 [0.0 - 0.0] | 0.0 [0.0 - 0.0] | 0.0 [0.0 - 0.0] | 0.0 [0.0 - 0.0] | 0.0 [0.0 - 0.0] | 0.0 [0.0 - 0.0] | 0.0 [0.0 - 0.0] | 0.0 [0.0 - 0.0] | 0.0 [0.0 - 0.0] | 0.0 [0.0 - 0.0] | 0.0 [0.0 - 0.0] | 0.0 [0.0 - 0.0] | 0.0 [0.0 - 0.0] |
| THC | 0.0 [0.0 - 0.0] | 3.0 [2.0 - 8.0] | 5.1 [2.7 - 8.2] | 4.0 [1.5 - 8.0] | 6.2 [2.4 - 8.5] | 3.0 [1.3 - 8.3] | 2.5 [1.0 - 6.1] | 1.2 [0.0 - 3.7] | 0.0 [0.0 - 1.3] | 0.0 [0.0 - 0.5] | 0.0 [0.0 - 0.0] | 0.0 [0.0 - 0.0] | 0.0 [0.0 - 0.0] | 0.0 [0.0 - 0.0] |
| P | 1.00 | <0.001 | <0.001 | <0.001 | <0.001 | 0.002 | 0.035 | 1.00 | 1.00 | 1.00 | 1.00 | 1.00 | 1.00 | 1.00 |
| **Irritation** | | | | | | | | | | | | | | |
| Placebo | 0.0 [0.0 - 0.0] | 0.0 [0.0 - 0.0] | 0.0 [0.0 - 0.0] | 0.0 [0.0 - 0.0] | 0.0 [0.0 - 0.0] | 0.0 [0.0 - 0.0] | 0.0 [0.0 - 0.0] | 0.0 [0.0 - 0.0] | 0.0 [0.0 - 0.0] | 0.0 [0.0 - 0.0] | 0.0 [0.0 - 0.0] | 0.0 [0.0 - 0.0] | 0.0 [0.0 - 0.0] | 0.0 [0.0 - 0.0] |
| THC | 0.0 [0.0 - 0.0] | 1.0 [0.0 - 2.3] | 2.0 [1.0 - 3.2] | 2.5 [1.0 - 6.0] | 2.0 [0.0 - 2.9] | 2.0 [0.0 - 6.0] | 1.0 [0.0 - 4.4] | 1.0 [0.0 - 1.7] | 0.0 [0.0 - 1.1] | 0.0 [0.0 - 2.0] | 0.0 [0.0 - 1.0] | 0.0 [0.0 - 0.0] | 0.0 [0.0 - 0.0] | 0.0 [0.0 - 0.0] |
| P | 1.00 | 1.00 | 1.00 | 0.064 | 0.437 | 0.139 | 0.832 | 1.00 | 1.00 | 1.00 | 1.00 | 1.00 | 1.00 | 1.00 |
| **Sleepiness** | | | | | | | | | | | | | | |
| Placebo | 2.5 [1.3 - 3.3] | 2.5 [1.3 - 3.7] | 2.5 [1.2 - 3.4] | 0.5 [0.0 - 2.1] | 1.0 [0.0 - 2.7] | 1.5 [0.2 - 2.4] | 0.5 [0.0 - 1.7] | 1.5 [0.3 - 2.8] | 1.0 [1.0 - 1.7] | 1.0 [1.0 - 1.0] | 1.0 [0.3 - 1.7] | 0.0 [0.0 - 0.7] | 0.0 [0.0 - 0.8] | 1.0 [0.0 - 1.0] |
| THC | 2.0 [1.0 - 3.0] | 7.5 [6.0 - 8.0] | 8.0 [6.8 - 9.0] | 8.0 [7.0 - 9.5] | 9.0 [8.0 - 10.0] | 9.0 [7.5 - 10.0] | 9.0 [7.0 - 9.8] | 9.0 [6.7 - 9.8] | 8.0 [7.0 - 9.0] | 6.7 [5.2 - 9.0] | 7.0 [4.0 - 8.0] | 6.0 [2.0 - 8.0] | 2.0 [0.0 - 5.0] | 2.0 [0.0 - 4.0] |
| P | 1.00 | 0.034 | 0.012 | <0.001 | <0.001 | <0.001 | <0.001 | <0.001 | <0.001 | <0.001 | <0.001 | 0.004 | 1.00 | 1.00 |
| **Strange thoughts/ ideas/ mood** | | | | | | | | | | | | | | |
| Placebo | 0.0 [0.0 - 0.0] | 0.0 [0.0 - 0.0] | 0.0 [0.0 - 0.0] | 0.0 [0.0 - 0.0] | 0.0 [0.0 - 0.0] | 0.0 [0.0 - 0.0] | 0.0 [0.0 - 0.0] | 0.0 [0.0 - 0.0] | 0.0 [0.0 - 0.0] | 0.0 [0.0 - 0.0] | 0.0 [0.0 - 0.0] | 0.0 [0.0 - 0.0] | 0.0 [0.0 - 0.0] | 0.0 [0.0 - 0.0] |
| THC | 0.0 [0.0 - 0.0] | 7.0 [3.7 - 9.0] | 6.5 [5.0 - 10.0] | 5.2 [3.0 - 9.6] | 7.0 [3.5 - 10.0] | 6.0 [3.5 - 9.5] | 4.0 [1.5 - 8.0] | 3.0 [1.2 - 5.0] | 2.0 [0.7 - 4.0] | 0.3 [0.0 - 2.0] | 0.0 [0.0 - 1.0] | 0.0 [0.0 - 0.0] | 0.0 [0.0 - 0.0] | 0.0 [0.0 - 0.0] |
| P | 1.00 | <0.001 | <0.001 | <0.001 | <0.001 | <0.001 | <0.001 | 0.058 | 1.00 | 1.00 | 1.00 | 1.00 | 1.00 | 1.00 |
| **Tenseness/ aggressiveness** | | | | | | | | | | | | | | |
| Placebo | 0.5 [0.0 - 2.5] | 0.5 [0.0 - 1.0] | 0.0 [0.0 - 0.7] | 0.0 [0.0 - 0.0] | 0.0 [0.0 - 0.0] | 0.0 [0.0 - 0.0] | 0.0 [0.0 - 0.0] | 0.0 [0.0 - 0.0] | 0.0 [0.0 - 0.0] | 0.0 [0.0 - 0.7] | 0.0 [0.0 - 0.0] | 0.0 [0.0 - 0.0] | 0.0 [0.0 - 0.0] | 0.0 [0.0 - 0.0] |
| THC | 0.0 [0.0 - 2.0] | 4.5 [2.0 - 7.0] | 5.4 [2.7 - 7.4] | 5.0 [3.0 - 7.4] | 2.0 [1.0 - 6.0] | 2.0 [0.5 - 6.2] | 2.0 [1.0 - 4.8] | 1.8 [0.0 - 3.0] | 1.3 [0.0 - 2.3] | 0.0 [0.0 - 2.0] | 0.0 [0.0 - 1.0] | 0.0 [0.0 - 0.0] | 0.0 [0.0 - 0.0] | 0.0 [0.0 - 0.0] |
| P | 1.000 | 0.907 | 0.002 | 0.010 | 0.112 | 0.444 | 0.275 | 1.00 | 1.00 | 1.00 | 1.00 | 1.00 | 1.00 | 1.00 |

Supplementary Table 1B: Somatic effects of THC as assessed by questionnaire 1 over time. Data represent measurements on a visual analog scale from 0 to 10 and are expressed as median (IQR).

|  | 0 min | 10 min | 30 min | 45 min | 60 min | 75 min | 90 min | 120 min | 150 min | 180 min | 240 min | 300 min | 1440 min (24h) | 2880 min (48h) |
| --- | --- | --- | --- | --- | --- | --- | --- | --- | --- | --- | --- | --- | --- | --- |
| **Pooled somatic effects** | | | | | | | | | | | | | | |
| Placebo | 0.0 [0.0 - 0.6] | 0.0 [0.0 - 0.3] | 0.0 [0.0 - 0.3] | 0.0 [0.0 - 0.3] | 0.0 [0.0 - 0.3] | 0.0 [0.0 - 0.3] | 0.0 [0.0 - 0.2] | 0.1 [0.0 - 0.4] | 0.1 [0.0 - 0.4] | 0.1 [0.0 - 0.2] | 0.0 [0.0 - 0.2] | 0.1 [0.0 - 0.2] | 0.0 [0.0 - 0.0] | 0.0 [0.0 - 0.0] |
| THC | 0.0 [0.0 - 0.4] | 6.0 [3.8 - 7.0] | 5.9 [3.7 - 6.8] | 5.0 [3.4 - 6.9] | 5.1 [3.3 - 5.6] | 4.8 [3.2 - 5.8] | 4.1 [2.4 - 5.9] | 3.2 [1.8 - 4.9] | 2.4 [1.3 - 4.3] | 1.6 [0.8 - 2.4] | 0.8 [0.4 - 2.2] | 1.0 [0.4 - 1.4] | 0.0 [0.0 - 0.4] | 0.0 [0.0 - 0.4] |
| P | 1.00 | <0.001 | <0.001 | <0.001 | <0.001 | <0.001 | <0.001 | <0.001 | <0.001 | 0.006 | 0.258 | 1.00 | 1.00 | 1.00 |
| **Cardiac problems (e.g. fast pulse)** | | | | | | | | | | | | | | |
| Placebo | 0.0 [0.0 - 0.0] | 0.0 [0.0 - 0.0] | 0.0 [0.0 - 0.0] | 0.0 [0.0 - 0.0] | 0.0 [0.0 - 0.0] | 0.0 [0.0 - 0.0] | 0.0 [0.0 - 0.0] | 0.0 [0.0 - 0.0] | 0.0 [0.0 - 0.0] | 0.0 [0.0 - 0.0] | 0.0 [0.0 - 0.0] | 0.0 [0.0 - 0.0] | 0.0 [0.0 - 0.0] | 0.0 [0.0 - 0.0] |
| THC | 0.0 [0.0 - 0.0] | 6.5 [4.9 - 8.7] | 6.0 [2.9 - 9.0] | 5.0 [1.6 - 7.5] | 4.0 [2.2 - 8.0] | 4.0 [0.8 - 7.5] | 2.0 [0.5 - 7.2] | 1.0 [0.0 - 4.0] | 0.9 [0.0 - 1.9] | 0.0 [0.0 - 1.0] | 0.0 [0.0 - 0.0] | 0.0 [0.0 - 0.0] | 0.0 [0.0 - 0.0] | 0.0 [0.0 - 0.0] |
| P | 1.00 | <0.001 | <0.001 | 0.001 | 0.001 | 0.012 | 0.062 | 1.00 | 1.00 | 1.00 | 1.00 | 1.00 | 1.00 | 1.00 |
| **Difficulties in breathing** | | | | | | | | | | | | | | |
| Placebo | 0.0 [0.0 - 0.0] | 0.0 [0.0 - 0.0] | 0.0 [0.0 - 0.0] | 0.0 [0.0 - 0.0] | 0.0 [0.0 - 0.0] | 0.0 [0.0 - 0.0] | 0.0 [0.0 - 0.0] | 0.0 [0.0 - 0.0] | 0.0 [0.0 - 0.0] | 0.0 [0.0 - 0.0] | 0.0 [0.0 - 0.0] | 0.0 [0.0 - 0.0] | 0.0 [0.0 - 0.0] | 0.0 [0.0 - 0.0] |
| THC | 0.0 [0.0 - 0.0] | 3.0 [1.0 - 5.0] | 3.0 [1.0 - 5.2] | 3.0 [0.3 - 4.8] | 2.0 [0.5 - 5.5] | 2.0 [0.0 - 4.5] | 1.5 [0.0 - 3.0] | 1.0 [0.0 - 2.5] | 0.0 [0.0 - 1.0] | 0.0 [0.0 - 1.0] | 0.0 [0.0 - 0.0] | 0.0 [0.0 - 0.0] | 0.0 [0.0 - 0.0] | 0.0 [0.0 - 0.0] |
| P | 1.00 | 0.065 | 0.023 | 0.055 | 0.211 | 0.210 | 0.387 | 1.00 | 1.00 | 1.00 | 1.00 | 1.00 | 1.00 | 1.00 |
| **Dry mouth** | | | | | | | | | | | | | | |
| Placebo | 0.0 [0.0 - 1.5] | 0.0 [0.0 - 1.5] | 0.0 [0.0 - 1.5] | 0.0 [0.0 - 1.5] | 0.0 [0.0 - 1.5] | 0.0 [0.0 - 1.5] | 0.0 [0.0 - 0.7] | 0.0 [0.0 - 0.8] | 0.0 [0.0 - 0.8] | 0.0 [0.0 - 0.7] | 0.0 [0.0 - 0.0] | 0.0 [0.0 - 0.8] | 0.0 [0.0 - 0.0] | 0.0 [0.0 - 0.0] |
| THC | 0.0 [0.0 - 0.0] | 8.5 [6.8 - 10.0] | 8.5 [7.0 - 10.0] | 9.0 [7.0 - 10.0] | 8.0 [6.5 - 10.0] | 8.0 [7.0 - 10.0] | 8.6 [6.0 - 10.0] | 6.5 [5.5 - 9.2] | 6.5 [2.8 - 9.0] | 4.0 [2.0 - 6.0] | 2.0 [0.0 - 5.0] | 2.0 [0.0 - 4.0] | 0.0 [0.0 - 0.0] | 0.0 [0.0 - 0.0] |
| P | 1.00 | <0.001 | <0.001 | <0.001 | <0.001 | <0.001 | <0.001 | <0.001 | <0.001 | 0.091 | 1.00 | 1.00 | 1.00 | 1.00 |
| **Headache** | | | | | | | | | | | | | | |
| Placebo | 0.0 [0.0 - 0.0] | 0.0 [0.0 - 0.0] | 0.0 [0.0 - 0.0] | 0.0 [0.0 - 0.0] | 0.0 [0.0 - 0.0] | 0.0 [0.0 - 0.0] | 0.0 [0.0 - 0.0] | 0.0 [0.0 - 0.0] | 0.0 [0.0 - 0.0] | 0.0 [0.0 - 0.0] | 0.0 [0.0 - 0.0] | 0.0 [0.0 - 0.0] | 0.0 [0.0 - 0.0] | 0.0 [0.0 - 0.0] |
| THC | 0.0 [0.0 - 0.0] | 1.1 [0.0 - 6.0] | 2.4 [0.0 - 5.1] | 2.0 [0.0 - 4.0] | 1.4 [0.0 - 4.0] | 1.9 [0.0 - 3.8] | 1.0 [0.0 - 5.2] | 0.0 [0.0 - 4.0] | 0.5 [0.0 - 3.5] | 1.0 [0.0 - 2.0] | 0.0 [0.0 - 3.0] | 1.0 [0.0 - 2.0] | 0.0 [0.0 - 1.0] | 0.0 [0.0 - 0.0] |
| P | 1.00 | 1.00 | 0.597 | 1.00 | 1.00 | 1.00 | 1.00 | 1.00 | 1.00 | 1.00 | 1.00 | 1.00 | 1.00 | 1.00 |
| **Nausea** | | | | | | | | | | | | | | |
| Placebo | 0.0 [0.0 - 0.0] | 0.0 [0.0 - 0.0] | 0.0 [0.0 - 0.0] | 0.0 [0.0 - 0.0] | 0.0 [0.0 - 0.0] | 0.0 [0.0 - 0.0] | 0.0 [0.0 - 0.0] | 0.0 [0.0 - 0.0] | 0.0 [0.0 - 0.0] | 0.0 [0.0 - 0.0] | 0.0 [0.0 - 0.0] | 0.0 [0.0 - 0.0] | 0.0 [0.0 - 0.0] | 0.0 [0.0 - 0.0] |
| THC | 0.0 [0.0 - 0.0] | 9.0 [5.7 - 10.0] | 8.0 [5.5 - 10.0] | 7.0 [4.0 - 9.0] | 6.0 [4.5 - 9.0] | 7.0 [3.4 - 9.0] | 6.0 [2.5 - 7.8] | 6.0 [1.5 - 7.8] | 3.0 [1.0 - 6.0] | 2.0 [0.0 - 4.0] | 1.0 [0.0 - 3.0] | 1.0 [0.0 - 2.0] | 0.0 [0.0 - 1.0] | 0.0 [0.0 - 0.0] |
| P | 1.00 | <0.001 | <0.001 | <0.001 | <0.001 | <0.001 | <0.001 | <0.001 | 1.00 | 1.00 | 1.00 | 1.00 | 1.00 | 1.00 |

Supplementary Table 2: Psychotropic effects as assessed by questionnaire 2 over time. Data represent values on a 7-point Likert scale and are expressed as median (IQR).

|  | 0 min | 30 min | 90 min | 150 min | 300 min | 1440 min (24h) | 2880 min (48h) |
| --- | --- | --- | --- | --- | --- | --- | --- |
| **POSITIVE MOOD** | | | | | | | |
| **Activation** | | | | | | | |
| Placebo | 4.0 [2.1 - 4.4] | 3.5 [2.6 - 4.4] | 4.3 [2.1 - 5.3] | 3.7 [2.4 - 4.7] | 4.0 [3.6 - 4.8] | 4.3 [3.2 - 4.9] | 4.2 [3.2 - 4.9] |
| THC | 4.0 [3.5 - 4.5] | 1.0 [0.5 - 2.0] | 2.0 [0.0 - 2.5] | 2.0 [1.5 - 2.5] | 2.5 [1.5 - 3.0] | 4.0 [3.0 - 4.5] | 4.2 [4.0 - 4.5] |
| P | 1.00 | 0.004 | <0.001 | 0.631 | 0.094 | 1.00 | 1.00 |
| **Balance** | | | | | | | |
| Placebo | 4.2 [3.6 - 4.5] | 4.5 [3.4 - 4.9] | 5.0 [3.9 - 5.0] | 4.8 [3.4 - 5.7] | 5.3 [5.0 - 5.9] | 4.3 [3.3 - 4.5] | 4.5 [3.4 - 5.2] |
| THC | 4.0 [3.0 - 5.0] | 1.0 [0.0 - 1.5] | 2.0 [1.0 - 3.0] | 3.0 [2.0 - 4.0] | 3.5 [3.0 - 4.5] | 4.0 [3.5 - 4.5] | 4.2 [3.5 - 5.0] |
| P | 1.000 | <0.001 | <0.001 | 0.156 | 0.189 | 1.000 | 1.000 |
| **Lifted Mood** | | | | | | | |
| Placebo | 4.0 [4.0 - 4.4] | 3.5 [3.0 - 5.1] | 3.8 [2.3 - 5.2] | 5.0 [4.1 - 5.5] | 4.8 [4.5 - 5.0] | 4.3 [4.0 - 4.5] | 4.0 [2.9 - 4.7] |
| THC | 4.0 [3.0 - 4.0] | 1.5 [0.0 - 2.5] | 2.0 [1.0 - 3.5] | 3.0 [2.0 - 3.5] | 3.0 [2.0 - 3.0] | 4.0 [3.0 - 4.5] | 4.0 [3.4 - 4.5] |
| P | 1.00 | <0.001 | 0.099 | 0.009 | 0.073 | 1.00 | 1.00 |
| **NEGATIVE MOOD** | | | | | | | |
| **Anxiety** | | | | | | | |
| Placebo | 0.7 [0.0 - 1.5] | 0.3 [0.0 - 0.5] | 0.0 [0.0 - 0.4] | 0.5 [0.0 - 1.4] | 0.0 [0.0 - 0.0] | 0.0 [0.0 - 0.0] | 0.0 [0.0 - 0.0] |
| THC | 1.0 [0.0 - 2.0] | 4.0 [2.5 - 5.0] | 2.0 [0.5 - 4.0] | 2.0 [0.0 - 2.5] | 0.0 [0.0 - 1.0] | 0.0 [0.0 - 1.0] | 0.0 [0.0 - 0.5] |
| P | 1.00 | <0.001 | 0.02 | 1.00 | 1.00 | 1.00 | 1.00 |
| **Bad Mood** | | | | | | | |
| Placebo | 0.0 [0.0 - 0.8] | 0.0 [0.0 - 0.4] | 0.0 [0.0 - 0.7] | 0.0 [0.0 - 0.0] | 0.3 [0.0 - 0.5] | 0.0 [0.0 - 0.0] | 0.0 [0.0 - 0.0] |
| THC | 0.0 [0.0 - 0.5] | 2.5 [2.0 - 4.0] | 1.0 [0.0 - 3.0] | 1.0 [0.0 - 1.5] | 0.0 [0.0 - 1.0] | 0.0 [0.0 - 0.5] | 0.0 [0.0 - 1.0] |
| P | 1.000 | <0.001 | 1.00 | 1.00 | 1.00 | 1.00 | 1.00 |
| **Deactivation** | | | | | | | |
| Placebo | 1.8 [1.1 - 2.0] | 1.5 [1.1 - 1.9] | 0.8 [0.1 - 1.7] | 1.3 [0.6 - 2.3] | 0.3 [0.0 - 0.9] | 0.5 [0.1 - 0.5] | 1.0 [0.3 - 1.0] |
| THC | 2.0 [1.0 - 2.5] | 4.5 [3.0 - 5.5] | 4.5 [3.0 - 5.5] | 4.5 [4.0 - 5.5] | 3.5 [2.0 - 5.0] | 1.5 [0.5 - 2.5] | 1.5 [0.4 - 2.1] |
| P | 1.00 | 0.305 | 0.010 | 0.009 | <0.001 | 1.00 | 1.00 |
| **Depression** | | | | | | | |
| Placebo | 0.2 [0.0 - 1.2] | 0.3 [0.0 - 0.5] | 0.0 [0.0 - 0.8] | 0.0 [0.0 - 0.0] | 0.0 [0.0 - 0.0] | 0.0 [0.0 - 0.0] | 0.0 [0.0 - 0.4] |
| THC | 0.0 [0.0 - 0.5] | 2.5 [2.0 - 3.0] | 1.5 [1.0 - 3.5] | 1.0 [0.0 - 2.0] | 0.0 [0.0 - 1.0] | 0.0 [0.0 - 0.0] | 0.0 [0.0 - 0.1] |
| P | 1.000 | 0.003 | 0.185 | 1.00 | 1.00 | 1.00 | 1.00 |
| **Excitation** | | | | | | | |
| Placebo | 2.5 [1.4 - 2.9] | 1.2 [0.3 - 1.5] | 0.5 [0.1 - 0.9] | 0.5 [0.1 - 0.5] | 0.3 [0.0 - 0.9] | 0.5 [0.1 - 0.9] | 0.3 [0.0 - 0.9] |
| THC | 2.0 [0.5 - 3.0] | 4.5 [3.0 - 5.5] | 3.0 [1.5 - 4.0] | 2.0 [0.5 - 3.0] | 1.0 [0.0 - 1.5] | 0.5 [0.5 - 1.0] | 0.3 [0.0 - 1.0] |
| P | 1.00 | <0.001 | 0.087 | 1.00 | 1.00 | 1.00 | 1.00 |
| **Irritation** | | | | | | | |
| Placebo | 0.0 [0.0 - 0.4] | 0.0 [0.0 - 0.0] | 0.0 [0.0 - 0.0] | 0.0 [0.0 - 0.4] | 0.0 [0.0 - 0.0] | 0.0 [0.0 - 0.0] | 0.0 [0.0 - 0.0] |
| THC | 0.0 [0.0 - 0.0] | 1.0 [0.0 - 2.0] | 1.0 [0.0 - 1.5] | 0.0 [0.0 - 1.0] | 0.0 [0.0 - 0.0] | 0.0 [0.0 - 0.0] | 0.0 [0.0 - 0.1] |
| P | 1.000 | 0.129 | 1.00 | 1.00 | 1.00 | 1.00 | 1.00 |
| **OTHER** | | | | | | | |
| **Anhedonic reactivity** | | | | | | | |
| Placebo | 0.5 [0.1 - 0.9] | 0.3 [0.0 - 1.2] | 0.0 [0.0 - 0.4] | 0.0 [0.0 - 0.4] | 0.2 [0.0 - 0.9] | 0.0 [0.0 - 0.4] | 0.0 [0.0 - 0.4] |
| THC | 0.0 [0.0 - 1.5] | 4.0 [2.0 - 5.5] | 2.0 [1.0 - 3.0] | 2.0 [1.0 - 3.0] | 1.0 [0.0 - 2.0] | 0.5 [0.0 - 1.0] | 0.0 [0.0 - 1.0] |
| P | 1.00 | 0.001 | 0.213 | 0.261 | 1.00 | 1.00 | 1.00 |
| **Capability** | | | | | | | |
| Placebo | 5.5 [4.2 - 6.0] | 4.5 [3.2 - 5.0] | 4.5 [2.5 - 5.7] | 4.5 [3.3 - 5.8] | 5.5 [4.3 - 6.0] | 4.5 [4.0 - 5.8] | 5.0 [5.0 - 5.0] |
| THC | 5.0 [4.0 - 5.0] | 1.0 [0.0 - 3.0] | 2.0 [0.5 - 2.5] | 3.0 [2.0 - 3.3] | 4.0 [3.0 - 4.0] | 5.0 [4.0 - 5.0] | 5.0 [4.0 - 5.0] |
| P | 1.00 | 0.009 | 0.004 | 0.113 | 0.244 | 1.00 | 1.00 |
| **Confidence** | | | | | | | |
| Placebo | 4.5 [3.2 - 5.0] | 4.7 [4.1 - 5.4] | 4.7 [2.6 - 5.4] | 5.0 [3.7 - 5.5] | 5.5 [4.4 - 5.9] | 5.3 [4.6 - 5.5] | 4.8 [3.3 - 5.5] |
| THC | 4.5 [4.0 - 5.0] | 2.0 [0.5 - 2.5] | 3.0 [2.0 - 3.5] | 3.5 [2.5 - 3.5] | 4.0 [3.0 - 4.5] | 4.5 [4.0 - 5.0] | 5.0 [4.0 - 5.0] |
| P | 1.00 | <0.001 | 0.058 | 0.064 | 0.144 | 1.00 | 1.00 |
| **Extraversion** | | | | | | | |
| Placebo | 4.0 [3.3 - 4.8] | 3.0 [2.2 - 4.5] | 4.0 [2.5 - 4.0] | 3.0 [2.2 - 3.7] | 4.0 [4.0 - 4.0] | 3.0 [3.0 - 3.8] | 4.0 [3.0 - 4.0] |
| THC | 4.0 [3.0 - 5.0] | 1.0 [0.0 - 2.0] | 2.0 [1.0 - 2.0] | 3.0 [2.0 - 3.0] | 3.0 [2.0 - 4.0] | 4.0 [3.0 - 5.0] | 4.0 [4.0 - 5.0] |
| P | 1.00 | 0.025 | 0.072 | 1.00 | 1.00 | 1.00 | 1.00 |
| **Hedonic reactivity** | | | | | | | |
| Placebo | 4.5 [4.1 - 5.2] | 4.5 [2.6 - 4.5] | 3.7 [2.3 - 4.5] | 4.3 [3.6 - 4.5] | 4.3 [4.0 - 5.3] | 4.5 [3.6 - 5.4] | 4.0 [3.1 - 5.6] |
| THC | 4.0 [3.0 - 5.0] | 2.0 [0.5 - 3.0] | 2.5 [1.5 - 3.5] | 3.0 [2.5 - 4.0] | 3.5 [3.0 - 4.5] | 4.5 [3.5 - 5.0] | 4.5 [4.0 - 5.0] |
| P | 1.00 | 0.045 | 1.00 | 0.818 | 0.953 | 1.00 | 1.00 |
| **Introversion** | | | | | | | |
| Placebo | 0.5 [0.0 - 1.0] | 0.0 [0.0 - 0.0] | 0.0 [0.0 - 0.0] | 0.0 [0.0 - 0.7] | 0.0 [0.0 - 0.0] | 0.0 [0.0 - 0.0] | 1.0 [0.0 - 1.0] |
| THC | 1.0 [0.0 - 1.0] | 4.0 [3.0 - 5.0] | 4.0 [2.5 - 5.0] | 3.0 [2.0 - 3.0] | 1.0 [1.0 - 3.0] | 1.0 [0.0 - 1.0] | 0.5 [0.0 - 1.0] |
| P | 1.00 | <0.001 | <0.001 | 0.028 | 0.285 | 1.00 | 1.00 |
| Physical wellbeing | | | | | | | |
| Placebo | 3.0 [2.6 - 3.0] | 3.0 [2.6 - 3.0] | 3.0 [2.6 - 3.4] | 3.0 [2.6 - 3.0] | 2.7 [2.5 - 3.0] | 2.7 [2.1 - 3.0] | 2.3 [2.0 - 2.9] |
| THC | 3.0 [2.5 - 3.0] | 2.5 [0.0 - 3.5] | 2.5 [1.5 - 3.0] | 3.0 [2.5 - 3.0] | 3.0 [2.5 - 3.0] | 3.0 [2.5 - 3.0] | 3.0 [2.5 - 3.0] |
| P | 1.00 | 0.080 | 0.273 | 1.00 | 1.00 | 1.00 | 0.873 |
| **Vitality** | | | | | | | |
| Placebo | 5.0 [5.0 - 5.0] | 5.0 [2.7 - 5.0] | 5.0 [4.3 - 5.0] | 5.0 [4.2 - 5.0] | 5.0 [5.0 - 5.0] | 5.0 [4.2 - 5.0] | 5.0 [5.0 - 5.0] |
| THC | 5.0 [4.0 - 5.0] | 2.0 [1.0 - 3.0] | 3.0 [2.0 - 4.5] | 4.0 [3.0 - 4.0] | 4.0 [3.0 - 5.0] | 5.0 [4.0 - 5.0] | 5.0 [4.7 - 5.3] |
| P | 1.00 | 0.027 | 0.844 | 0.515 | 1.00 | 1.00 | 1.00 |
