# Supplementary figures and images for: Quantification and time course of subjective psychotropic and somatic effects of tetrahydrocannabinol – a prospective, single-blind, placebo-controlled exploratory trial in healthy volunteers
Source: BMC Psychiatry. 2024 Dec 18;24:902. doi: 10.1186/s12888-024-06338-2 (PMC11654089; doi:10.1186/s12888-024-06338-2)

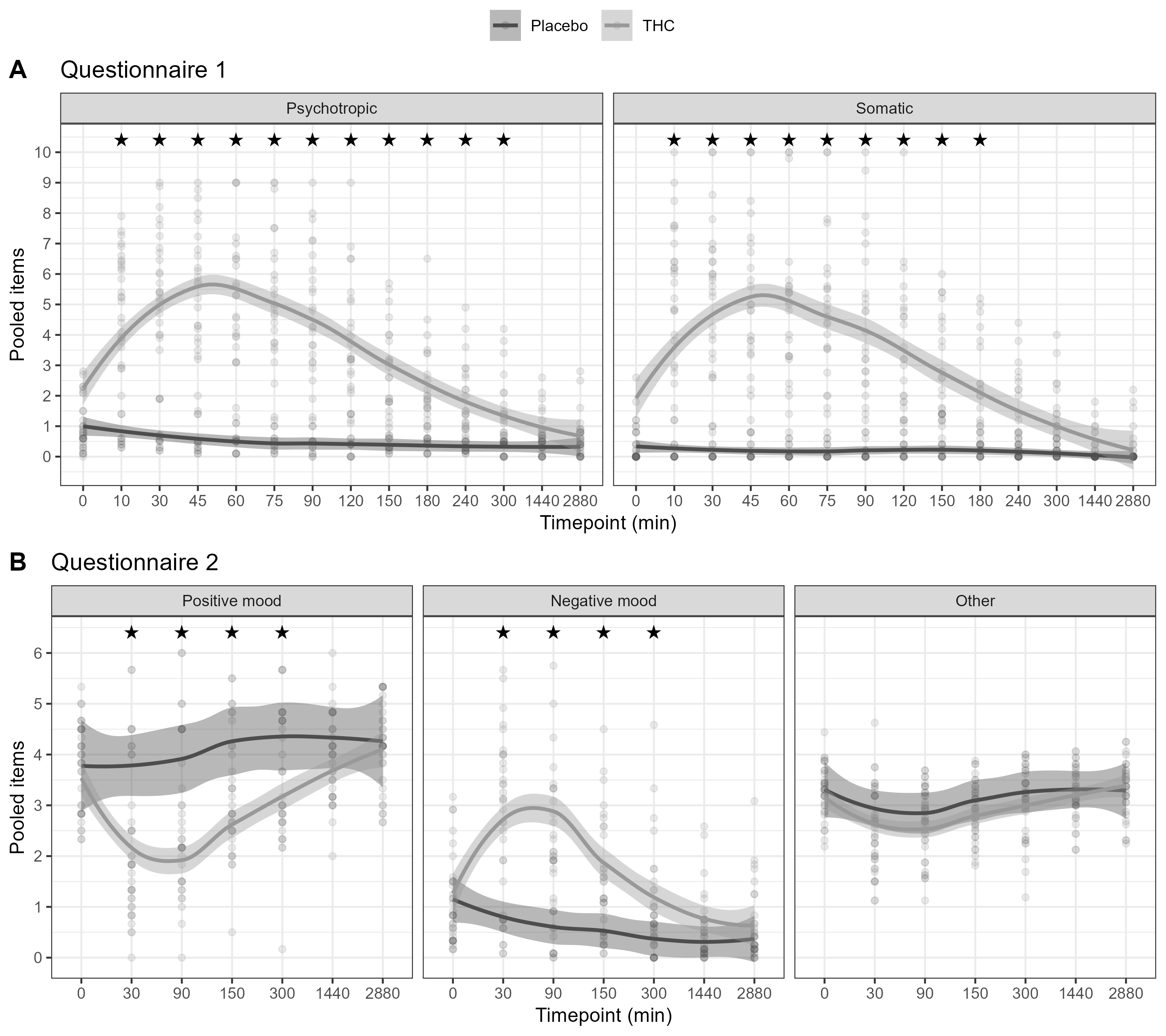

Supplement: Supplementary file 2 — Supplementary Material 2: Fig. 1: Psychotropic (A) and somatic (B) effects as assessed by questionnaire 1. Data are median and IQR. Missing values are imputed using the timepoint-wise median value. The asterisks (*) mark all statistically significant differences between placebo and THC groups. [file 12888_2024_6338_MOESM2_ESM.png]

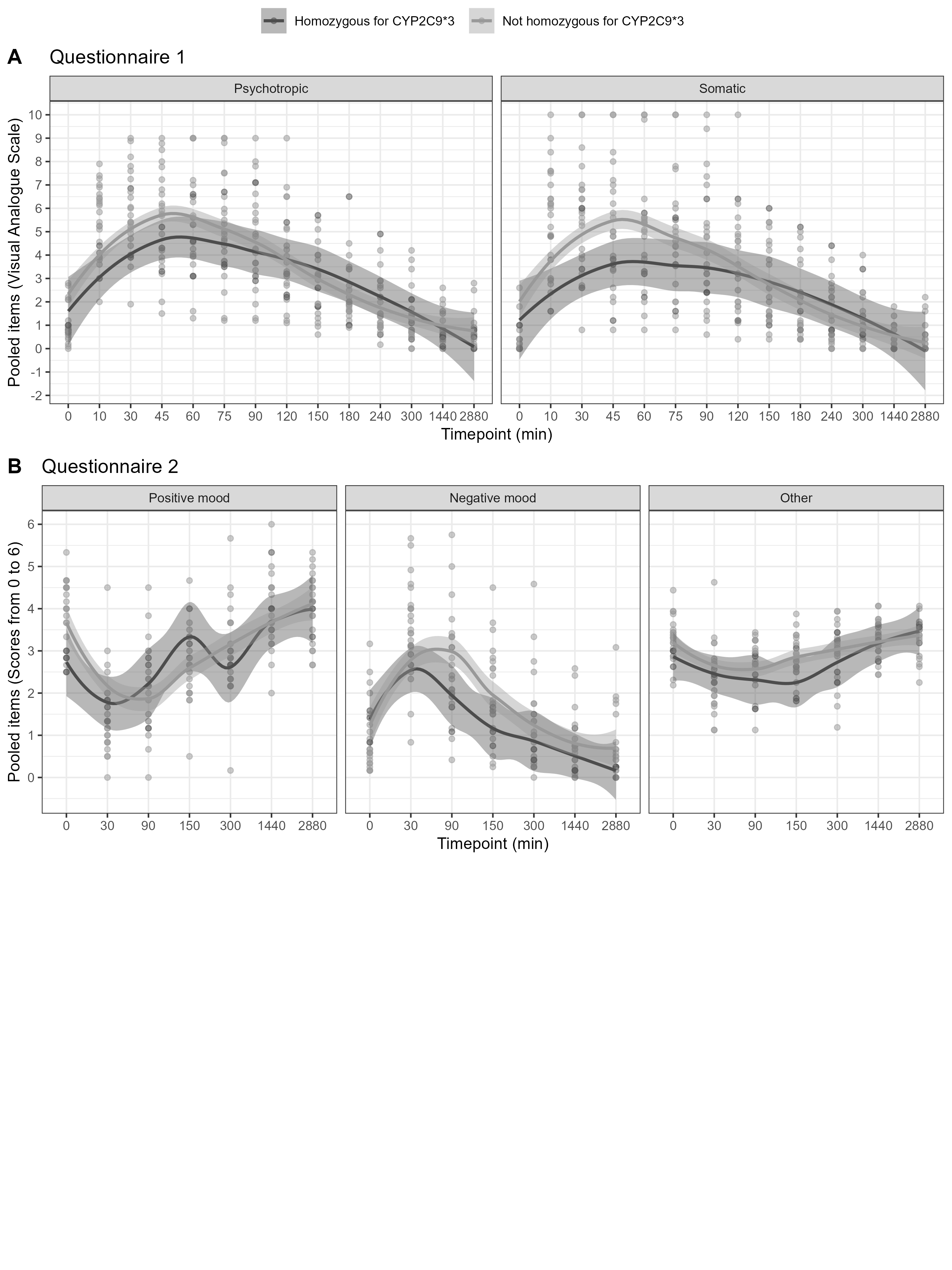

Supplement: Supplementary file 3 — Supplementary Material 3: Fig. 2: Effects of IV THC as assessed by questionnaire 2, summarized by the different subdimensions of the global dimensions “positive mood” (A), “negative mood” (B) and “other” (C). Data are median and IQR. Missing values are imputed using the timepoint-wise median value. The asterisks (*) mark all statistically significant differences between placebo and THC groups. [file 12888_2024_6338_MOESM3_ESM.png]

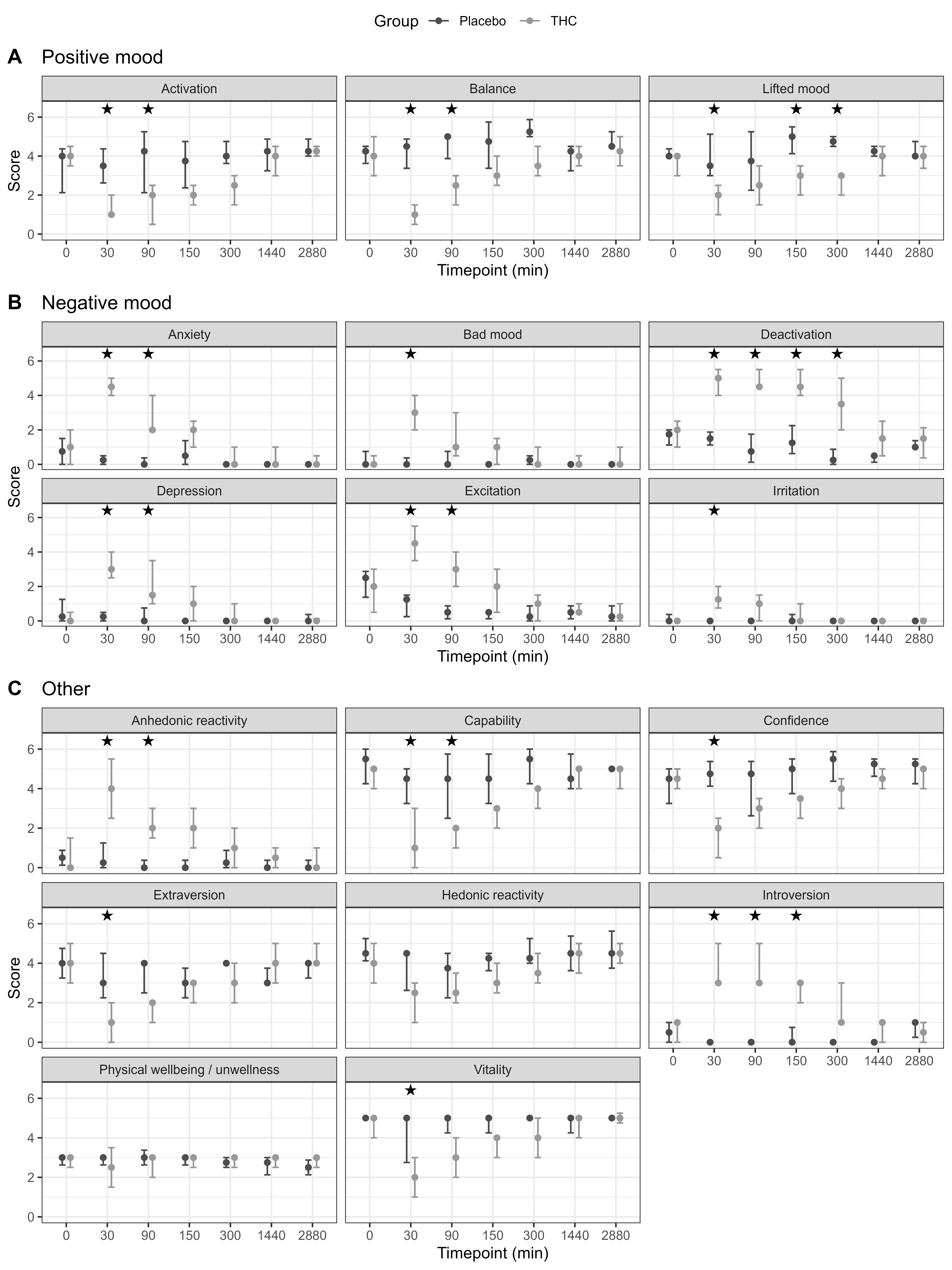

Supplement: Supplementary file 4 — Supplementary Material 4: Fig. 3: Global dimensions of psychotropic and somatic effects to assess peak effect and duration of effects. Missing values are imputed using the timepoint-wise median value. Asterisks (*) are used to mark all statistically significant differences. [file 12888_2024_6338_MOESM4_ESM.png]

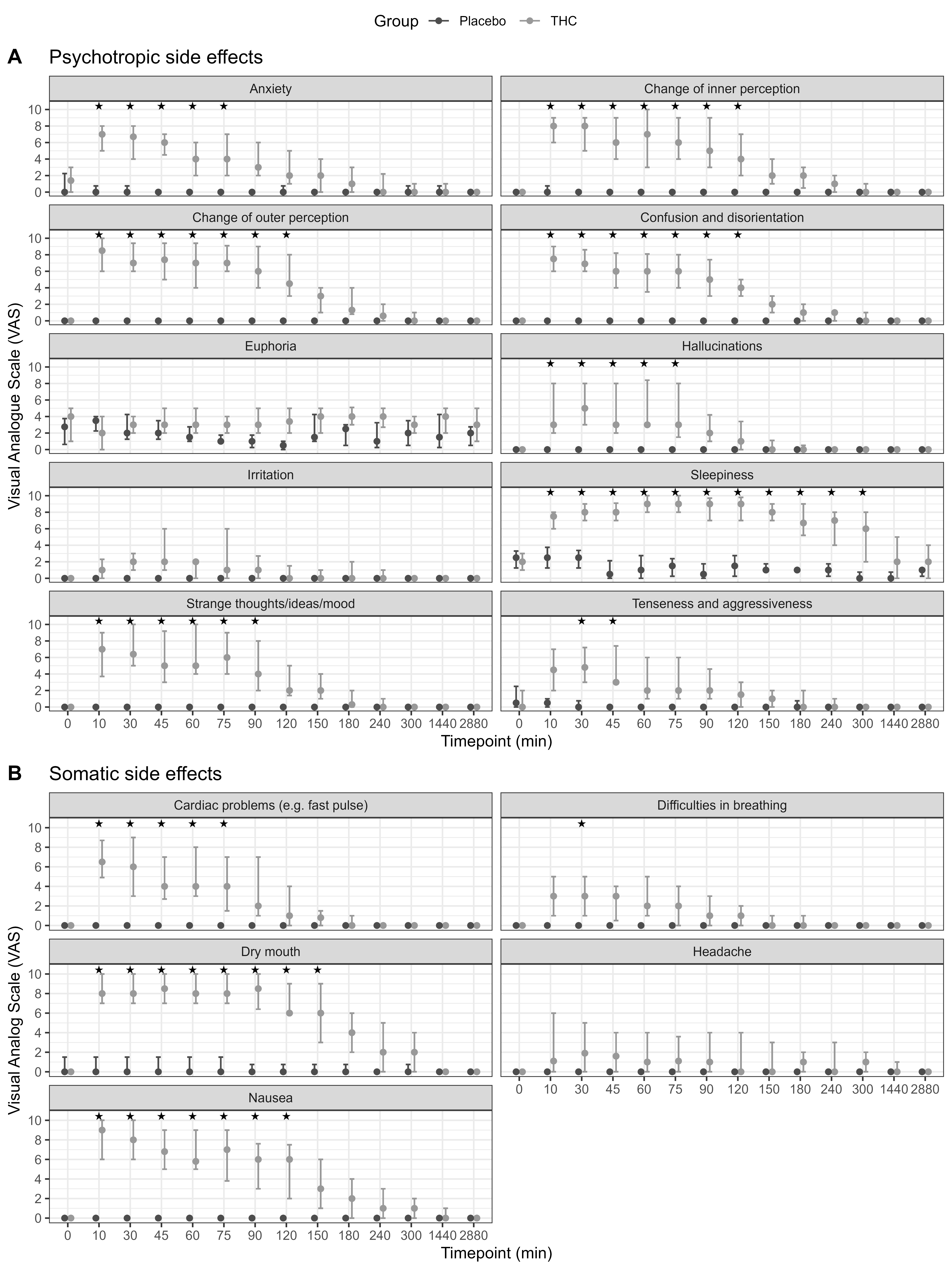

Supplement: Supplementary file 5 — Supplementary Material 5: Fig. 4: Graphical analysis of THC effects in volunteers homozygous (n = 3) or not homozygous (n = 22) for CYP2C9*3, receiving IV THC. Missing values are imputed using the timepoint-wise median value. No statistical significance testing was performed due to the small sample size. [file 12888_2024_6338_MOESM5_ESM.png]
